# Tissue Distribution of [^14^C]-Lefamulin into the Urogenital Tract in Rats

**DOI:** 10.1128/aac.00355-22

**Published:** 2022-07-11

**Authors:** Wolfgang W. Wicha, Claire Henson, Kathryn Webbley, Steven P. Gelone

**Affiliations:** a Nabriva Therapeutics GmbH, Vienna, Austria; b Pharmaron UK Ltd., Rushden, United Kingdom; c Nabriva Therapeutics US, Inc., Fort Washington, Pennsylvania, USA

**Keywords:** antibacterial agents, lefamulin, tissue distribution, pleuromutilin, sexually transmitted infection

## Abstract

Lefamulin, a semisynthetic pleuromutilin antibiotic approved in the United States, Canada, and Europe for intravenous and oral treatment of community-acquired bacterial pneumonia, is highly active *in vitro* against bacterial pathogens that cause sexually transmitted infections (STIs), including multidrug-resistant strains of Neisseria gonorrhoeae, Chlamydia trachomatis, and Mycoplasma genitalium. This nonclinical study used quantitative whole-body autoradiography (QWBA) and qualitative tape-transfer microautoradiography (MARG) to investigate lefamulin distribution into urogenital tract tissues down to a cellular level in male and female rats. A single intravenous dose (30 mg/kg) of [^14^C]-lefamulin was administered to 3 male and 3 female Sprague-Dawley rats. At 0.5, 6, and 24 h post dose, rats were euthanized and [^14^C]-lefamulin distribution was investigated using QWBA and MARG of sagittal planes. [^14^C]-lefamulin was well distributed throughout the carcasses of male and female rats, with the highest concentrations observed in male bulbourethral gland, urethra, prostate in female clitoral gland, uterus (particularly endometrium), and ovary. In these areas, concentrations were similar to or higher than those observed in the lungs. Concentrations peaked at 0.5 h post dose, remaining detectable in the urogenital tract up to 24 h post dose. [^14^C]-lefamulin in rats showed rapid, homogeneous distribution into urogenital tissues down to a cellular level, with high tissue:blood ratios in tissues relevant to STI treatment. These results, and the potent *in vitro* activity of lefamulin against multidrug-resistant bacteria known to cause STIs, will help inform further assessment of lefamulin, including potential clinical evaluation for treatment of STIs.

## INTRODUCTION

Sexually transmitted infections (STIs) have a major impact on reproductive health (1). The World Health Organization estimates that worldwide 1 million STIs are acquired each day (1). The incidence rates and causes of STIs vary by geographic region; however, in the United States, the most common bacterial causes of STIs are Chlamydia trachomatis and Neisseria gonorrhoeae, for which incidence rates appear to be increasing (2). STIs can cause serious health problems if left untreated or undertreated (2–6). In women, for example, gonorrhea and chlamydia can lead to pelvic inflammatory disease, which can result in chronic pelvic pain, ectopic pregnancy, and infertility (3, 5). Mycoplasma genitalium, another STI-associated pathogen, causes urethritis in men and has also been associated with cervicitis, pelvic inflammatory disease, infertility, spontaneous abortions, and preterm births in women (4, 7). Although treatment options are available for STIs, antibiotic resistance is a persistent problem that is becoming increasingly common for M. genitalium (8) and particularly N. gonorrhoeae (5). The US Centers for Disease Control and Prevention has estimated that 51.3% of N. gonorrhoeae isolates are resistant to at least one antibiotic (2). In some settings, approximately 50% of M. genitalium strains are resistant to azithromycin (4), and resistance to moxifloxacin has also been reported (9).

Lefamulin is the first pleuromutilin antibiotic approved in the United States, Canada, and Europe for intravenous (IV) and oral use in adults with community-acquired bacterial pneumonia (CABP) (10–12). Lefamulin inhibits bacterial protein synthesis by binding selectively and specifically to the A- and P-sites of the peptidyl transferase center of the 50S ribosomal subunit (13). The Lefamulin Evaluation Against Pneumonia (LEAP) 1 and LEAP 2 phase 3 clinical trials in adults with CABP demonstrated the noninferiority of lefamulin versus moxifloxacin (12, 14).

Other studies have demonstrated potent *in vitro* activity of lefamulin against a variety of pathogens, including those commonly associated with STIs such as N. gonorrhoeae, C. trachomatis, and M. genitalium (15, 16). In these studies, lefamulin activity was unaffected by mechanisms of resistance to other antibiotic classes and no significant cross-resistance was detected. Additionally, pharmacokinetic studies have shown rapid penetration of lefamulin into the interstitial space of skeletal muscle, subcutaneous adipose tissue, and epithelial lining fluid, with exposure levels in the epithelial lining fluid 5.7-fold higher than the free fraction in plasma (17). The current study in rodents was conducted to investigate the tissue distribution of IV lefamulin to male and female urogenital tract tissue using quantitative whole-body autoradiography (QWBA) combined with tape transfer microautoradiography (MARG).

## RESULTS

### [^14^C]-Lefamulin distribution in the male urogenital tract.

**FIG 1:**
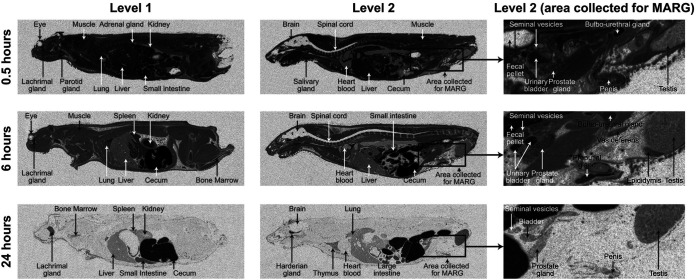
QWBA images of male rat showing distribution of [^14^C]-lefamulin at 0.5, 6, and 24 h after a single 30 mg/kg intravenous dose. Level 1 images were collected to investigate the distribution of radioactivity in the kidney; Level 2 images were collected to investigate the distribution of radioactivity in the reproductive organs/tissues associated with renal elimination. MARG samples were all approximately 5 cm in width. MARG indicates microautoradiographic; QWBA, quantitative whole-body autoradiography.

By 0.5 h following a single IV dose of 30 mg/kg, [^14^C]-lefamulin was well distributed throughout the male rat (Fig. 1), with the highest concentrations observed in glandular tissues (e.g., adrenal, bulbourethral, lachrymal, parotid, and salivary glands) and tissues involved in lefamulin elimination (e.g., liver, kidney, gastrointestinal tract, and urinary bladder). Radioactivity generally declined by 6 h post dose, at which point the highest [^14^C]-lefamulin concentrations were associated with the gastrointestinal tract (Fig. 1). By 24 h post dose, [^14^C]-lefamulin concentrations in the blood were below the limit of quantification (<0.062 μg equivalents/g) (Table 1).

**TABLE 1:** Concentration of [^14^C]-lefamulin in urogenital tissues of interest from male rats after a single 30 mg/kg intravenous dose^*a*^

Male tissue	μg equivalents^*b*^				Tissue:Blood ratio				Tissue:Lung ratio		
	0.5 h	6 h	24 h		0.5 h	6 h	24 h		0.5 h	6 h	24 h
Blood	3.4	0.7	BLQ		1.0	1.0	1.0		0.1	0.03	NC
Lung	26.5	20.5	0.4		7.8	29.3	>6.5		1.0	1.0	1.0
Bulbourethral gland	53.4	13.4	NS		15.8	20.5	NC		2.0	0.7	NC
Preputial gland	NS	15.4	9.8		NC	23.5	>158.1		NC	0.8	25.9
Prostate gland, whole	11.0	8.4	0.5		3.3	12.8	>8.1		0.4	0.4	1.3
Prostate gland, lumen	4.6	6.5	0.3		1.4	10.0	>4.8		0.2	0.3	0.8
Prostate gland, wall	14.4	12.5	0.7		4.3	19.0	>11.3		0.5	0.6	1.9
Seminal vesicles, whole	8.3	7.6	0.5		2.5	11.6	>8.1		0.3	0.4	1.2
Seminal vesicles, lumen	2.7	4.2	0.3		0.8	6.5	>4.8		0.1	0.2	0.8
Seminal vesicles, wall	16.7	10.7	0.6		5.0	16.3	>9.7		0.6	0.5	1.6
Testis	1.3	2.3	2.0		0.4	3.5	>32.3		0.1	0.1	5.2
Urethra	22.3	24.0	0.8		6.6	36.6	>12.9		0.8	1.2	2.1
Urinary bladder, whole	19.5	20.4	0.6		5.8	31.2	>9.7		0.7	1.0	1.6
Urinary bladder, contents	24.2	35.0	0.8		7.2	53.4	>12.9		0.9	1.7	2.2
Urinary bladder, wall	8.2	3.4	0.1		2.4	5.1	>1.6		0.3	0.2	0.2

a BLQ, below limit of accurate quantification (<0.062 μg equivalents/g); NA, not applicable; NC, not calculable (tissue is BLQ/NS at this time); NS, no sample, tissue not sectioned.

b Data are μg equivalents of lefamulin per g of tissue.

**FIG 2:**
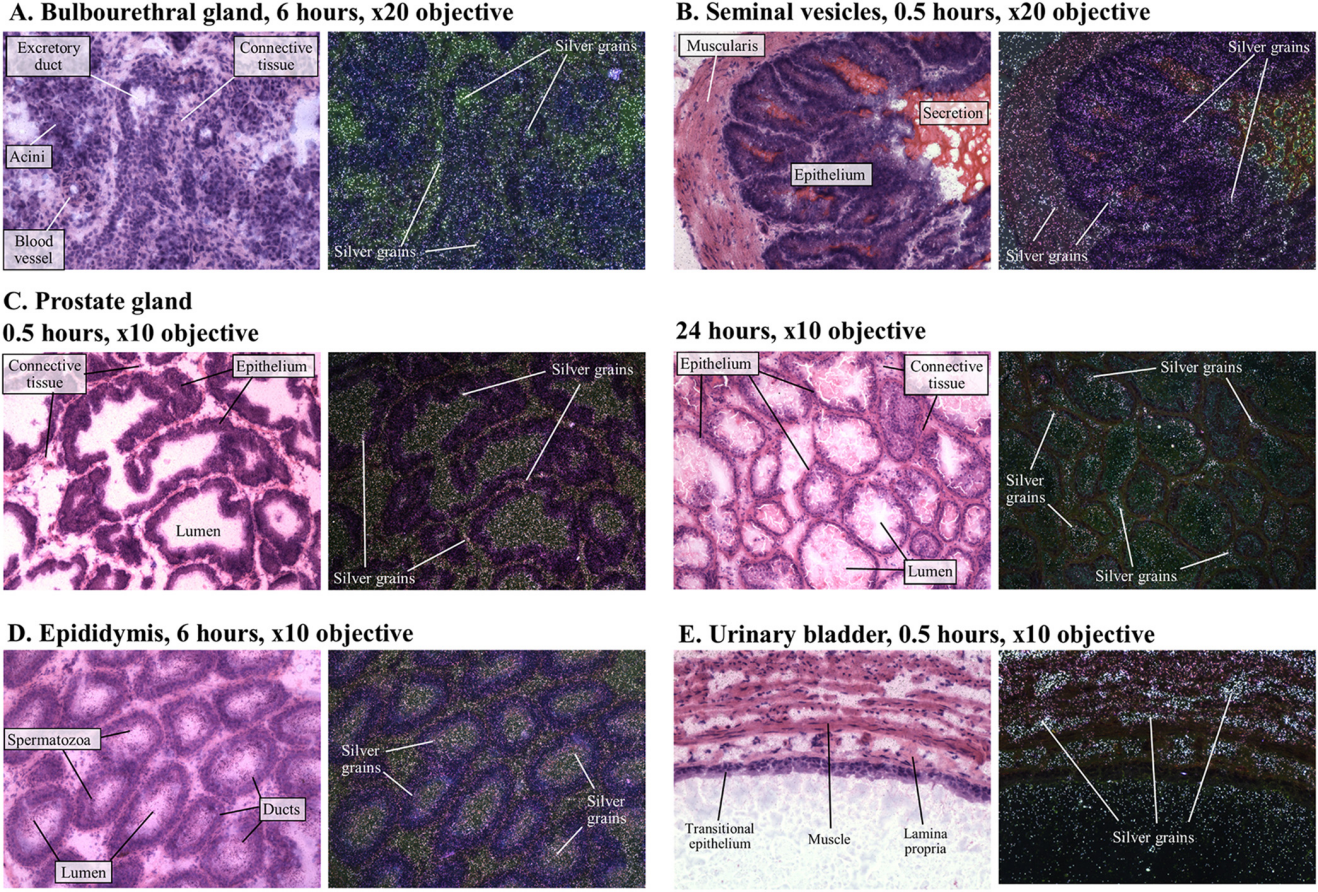
Microautoradiographs of individual tissues (A, bulbourethral gland; B, seminal vesicles; C, prostate gland; D, epididymis; and E, urinary bladder) within male rat showing distribution of [^14^C]-lefamulin after a single 30 mg/kg intravenous dose. Images are presented in both bright (hematoxylin and eosin) and dark field. Under bright field conditions, tissue histology can be viewed, and radioactivity (silver grains) shows as small black particles. Under dark field conditions, silver grains show up bright white against a dark, false-color background.

Within male urogenital tract tissues, concentrations of [^14^C]-lefamulin at 0.5 h post dose were higher in the bulbourethral gland and urethra than in other areas (Table 1; Fig. 1). Representative examples of MARG analyses in male tissues are shown in Fig. 2A to E. [^14^C]-lefamulin was homogeneously distributed across the bulbourethral gland (Fig. 2A), with concentrations peaking at 0.5 h and declining by 6 h post dose (Table 1; this tissue was not sampled for MARG analysis at 24 h). [^14^C]-lefamulin levels in the blood declined more rapidly than in the bulbourethral gland, resulting in increased tissue:blood ratios from 0.5 h to 6 h post dose (Table 1). Tissue:lung ratios demonstrated that [^14^C]-lefamulin levels in the bulbourethral gland were approximately double those observed in the lung at 0.5 h post dose and by 6 h post dose had diminished to levels below those in the lung. Concentrations in the urethra remained generally consistent from 0.5 h to 6 h post dose, contributing to tissue:lung ratios of approximately 1 and an approximately 6-fold increase in tissue:blood ratios. By 24 h post dose, lefamulin was nearly cleared from the urethra and no longer detectable in the blood, although the tissue:lung ratio reflected that [^14^C]-lefamulin levels remaining in the urethra were approximately double those remaining in the lung.

In the seminal vesicles and prostate gland, radioactivity was lower in the lumen than in the epithelial walls (Table 1; Fig. 2B and C), in which [^14^C]-lefamulin concentrations peaked at 0.5 h post dose, tissue:blood ratios peaked at 6 h post dose, and tissue:lung ratios peaked at 24 h post dose (Table 1). A similar pattern was observed in the preputial gland (data not shown), whereas radioactivity was generally homogenously distributed throughout the epididymis (Fig. 2D), urethra, vas deferens, and testes (data not shown). Urine does not transfer well to microscope slides using the tape-transfer technique, as demonstrated by the lack of radioactivity within the urinary bladder in Fig. 2E. The radioactivity observed within the bladder wall and epithelial folds is therefore likely due to residual or contaminating urine and the small amount of radioactivity observed within the epithelium itself diminishes toward the outer muscular wall of the bladder. Overall, [^14^C]-lefamulin concentrations in urogenital tissues were generally similar to those measured in the lung, with the exception of the preputial gland and testis, for which tissue:lung ratios had increased by approximately 32- and 52-fold at 24 h post dose compared with the 6-h post dose time point (Table 1).

### [^14^C]-Lefamulin distribution in the female urogenital tract.

**FIG 3:**
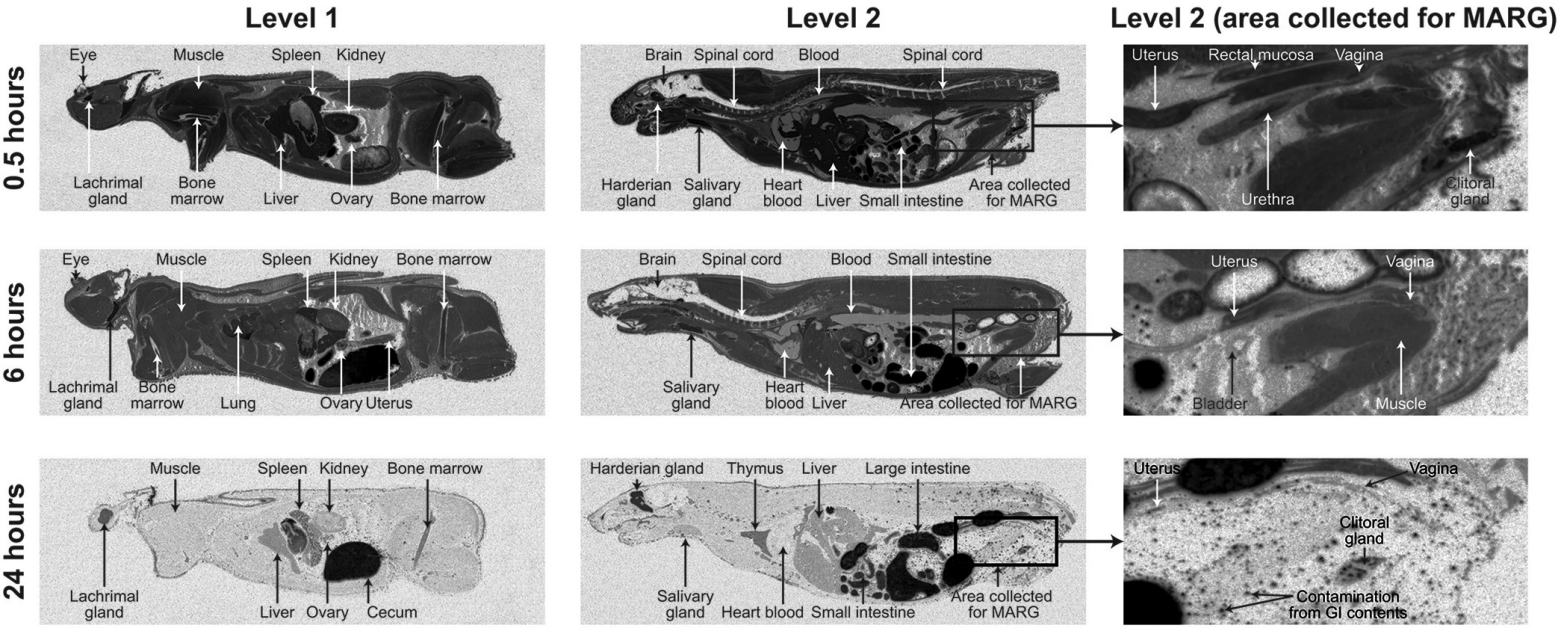
QWBA images of female rat showing distribution of [^14^C]-lefamulin at 0.5, 6, and 24 h after a single 30 mg/kg intravenous dose. Level 1 images were collected to investigate the distribution of radioactivity in the kidney and ovary/uterus; Level 2 images were collected to investigate the distribution of radioactivity in the reproductive organs/tissues associated with renal elimination. MARG samples were all approximately 5 cm in width. MARG indicates microautoradiographic; QWBA, quantitative whole-body autoradiography.

In female rats, [^14^C]-lefamulin was also well distributed throughout the tissues by 0.5 h post dose, with the highest concentrations observed in glandular tissue (e.g., Harderian, lachrymal, salivary, and clitoral) and tissues involved in elimination (e.g., liver, spleen, kidney, and contents of the small intestine) (Fig. 3). Radioactivity declined by 6 h post dose, at which point the highest [^14^C]-lefamulin concentrations were associated with the gastrointestinal tract. By 24 h, radioactivity in the blood was no longer detectable.

**FIG 4:**
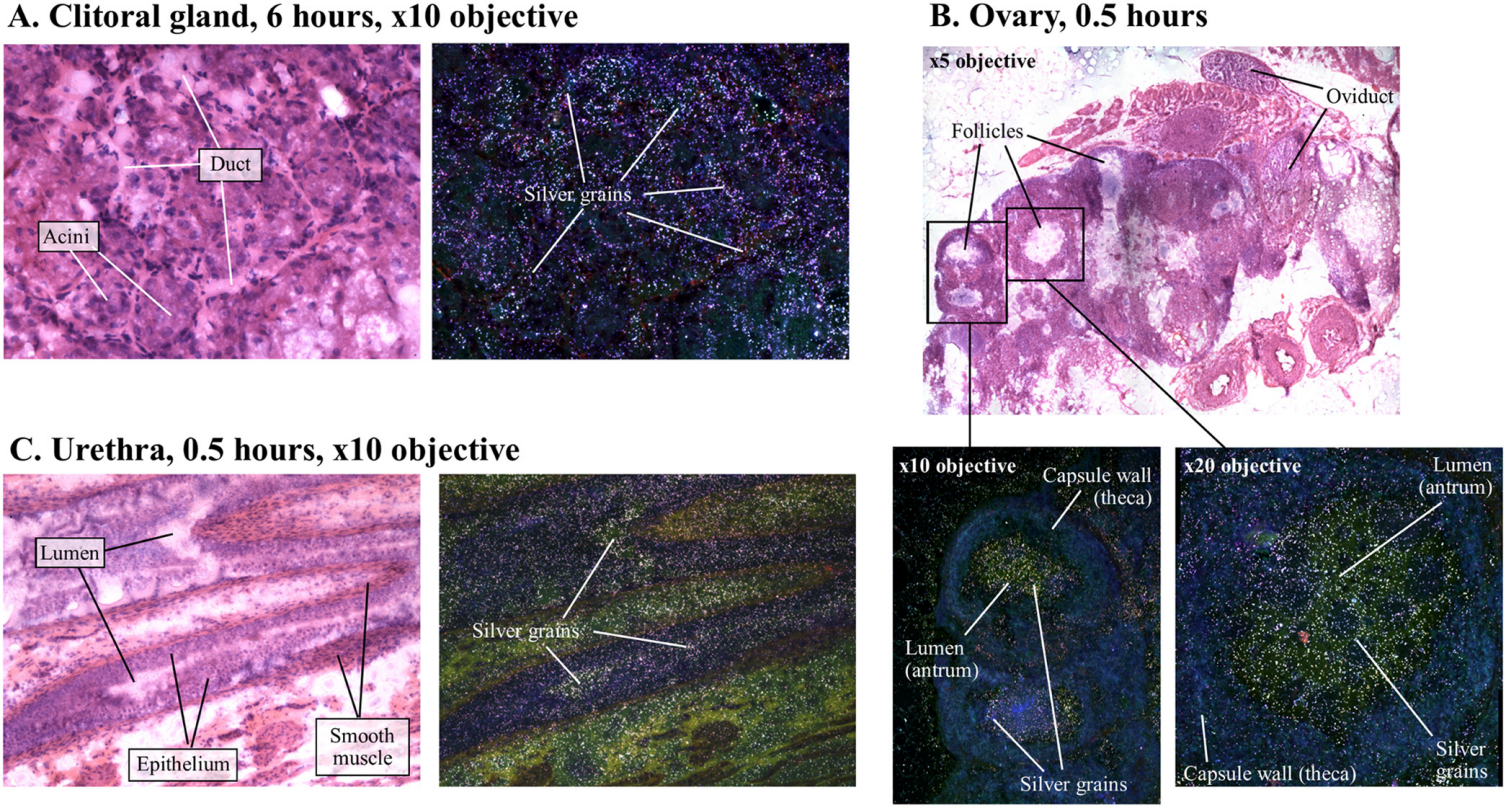
Microautoradiographs of individual tissues (A, clitoral; B, ovary; and C, urethra) within female rat showing distribution of [^14^C]-lefamulin after a single 30 mg/kg intravenous dose. Images are presented in both bright (hematoxylin and eosin) and dark field. Under bright field conditions, tissue histology can be viewed and radioactivity (silver grains) shows as small black particles. Under dark field conditions, silver grains show up bright white against a dark, false-color background.

In the female urogenital tract, the highest [^14^C]-lefamulin concentrations were observed in the clitoral gland, urethra, and uterus (Table 2). In the clitoral gland, distribution appeared to be homogeneous, with high concentrations in both the acini and sebaceous ducts at 0.5 h post dose, whereas radioactivity was concentrated in the ducts surrounding the acini at 6 and 24 h post dose (Fig. 4A). Radioactivity in the uterus was mainly observed in the endometrium (internal uterine mucosa) rather than the perimetrium or myometrium (data not shown). In the ovaries, [^14^C]-lefamulin was detected mainly in the follicular lumen, as seen at 0.5 h post dose in Fig. 4B, and a similar pattern of greater radioactivity within the lumen was observed in the urethra (Fig. 4C). In contrast, radioactivity within the vagina was homogenously spread throughout the lumen, epithelium, and smooth muscle wall at 0.5 and 6 h post dose (data not shown). In female rats, tissue concentrations of [^14^C]-lefamulin peaked at 0.5 h post dose in the urogenital tract, but concentrations decreased more rapidly in blood, leading to small (1.3- to 2.2-fold) increases in tissue:blood concentration ratios at 6 h post dose compared with the 0.5-h time point (Table 2). [^14^C]-lefamulin concentrations in urogenital tissues of female rats were generally similar to those measured in the lung; the greatest difference was observed for the clitoral gland, which had concentrations approximately 2- to 3-fold higher than in the lung at 0.5 and 24 h post dose, respectively.

**TABLE 2:** Concentration of [^14^C]-lefamulin in urogenital tissues of interest from female rats after a single 30 mg/kg intravenous dose administration^*a*^

Female tissue	μg equivalents^*b*^				Tissue:Blood ratio				Tissue:Lung ratio		
	0.5 h	6 h	24 h		0.5 h	6 h	24 h		0.5 h	6 h	24 h
Blood	5.6	1.1	BLQ		1.0	1.0	1.0		0.1	0.05	NC
Lung	39.5	22.4	0.8		7.1	20.4	>12.9		1.0	1.0	1.0
Clitoral gland	81.3	NS	2.4		14.5	NC	>38.7		2.1	NC	2.9
Ovary	27.3	9.8	0.5		4.9	8.9	>8.1		0.7	0.4	0.6
Urethra	32.0	NS	NS		5.7	NC	NC		0.8	NC	NC
Uterus, whole	31.9	8.4	0.6		5.7	7.7	>9.7		0.8	0.4	0.7
Endometrium	30.6	13.4	0.8		5.5	12.2	>12.9		0.8	0.6	1.0
Vagina	20.8	8.3	NS		3.7	7.6	NC		0.5	0.4	NC

a BLQ, below limit of accurate quantification (<0.062 μg equivalents/g); NA, not applicable; NC, not calculable (tissue is BLQ/NS at this time); NS, no sample, tissue not sectioned.

b Data are μg equivalents of lefamulin per g of tissue.

Because fat around the kidney and abdominal area interfered with emulsion coverage, particularly for females, affected sections were patchy and interpretation of radioactivity distribution was reduced for some animals. MARG data were therefore available only where the quality of slides allowed for interpretation.

## DISCUSSION

Following a single IV dose of 30 mg/kg, [^14^C]-lefamulin was distributed rapidly and in a homogeneous manner, down to the cellular level, in all STI-relevant urogenital tract tissues of male and female rats. Tissue concentrations of [^14^C]-lefamulin were generally highest at the earliest sampling time point of 0.5 h post dose and gradually decreased by 6 h, reaching levels below the limit of quantification by 24 h. Concentrations were similar to or exceeded those measured in the lung and were highest for the male bulbourethral gland, urethra, and urinary bladder and for the female clitoral gland, urethra, uterus, and ovaries. The high concentration of [^14^C]-lefamulin in the gastrointestinal tract 6 h post dose is consistent with the biliary excretion of lefamulin as the major route of elimination (10). The increase of some urogenital tract tissue:lung ratios from 0.5 h post dose to 24 h post dose suggests that lefamulin exposure in some parts of the urogenital tract may be extended compared with the lung. As previous studies in humans have demonstrated that lefamulin penetrates well into lung tissues, with high accumulation ratios compared with plasma (17, 18), the tissue:lung ratios obtained in this study for urogenital tissues reflecting lefamulin exposure at the site of STIs are probably more relevant for a potential pharmacokinetic/pharmacodynamic analysis than tissue:blood ratios. While the current study was focused specifically on distribution into the urogenital tract, previous studies utilizing QWBA methodology in rats quantified distribution in other tissues (19). Namely, with the exception of brain/spinal cord where lefamulin seemingly did not cross the blood-brain barrier under uninfected conditions, lefamulin concentrations in muscle, skin, lung, kidney, and liver were greater than those seen in blood already at 5 min, 30 min, and 6 h after intravenous administration. Taken together, these data help to fully characterize the distribution of lefamulin and could inform future clinical directions.

Because this study was limited by challenges with the MARG tape transfer system as described in the results section, the quality of some slides did not allow for interpretation. In addition, the use of thicker sections during MARG analyses and subsequent accumulation of silver grains did not allow for any attempts at semiquantitative estimations. Moreover, while interpretation of the results herein is limited by the small number of animals used and thus inability to estimate variability around observations, the consistency of findings over time suggests that, in rats, lefamulin appears to be rapidly distributed to tissues relevant to STIs, similar to the rapid penetration of lefamulin into epithelial lining fluid that was demonstrated previously (17, 18).

In the current study, a bolus injection of lefamulin into the tail vein led to systemic distribution throughout the urogenital tract. This finding supports the possibility that a similar distribution might be observed with IV administration in humans, although this hypothesis will require confirmation via future studies. Studies of cephalosporins administered intraperitoneally or orally in a female mouse genital tract infection model have, however, reported pharmacokinetics and pharmacodynamics consistent with current treatment recommendations for humans (20).

Several pathogens associated with STIs, particularly M. genitalium and N. gonorrhoeae, are gradually developing resistance to all available antibiotic therapies and new treatments are urgently needed (4, 16, 21). Lefamulin has been shown to be active against these bacterial pathogens, inducing *in vitro* inhibition of resistant strains of M. genitalium (MIC, 0.016–0.063 mg/L), N. gonorrhoeae (MIC_50/90_, 0.25/1 mg/L), and C. trachomatis (MIC_50/90_, 0.02/0.04 mg/L) (15, 16). The observations from the current study, suggesting rapid penetration of lefamulin to relevant areas of the rat urogenital tract comparable to the exposures in lung fluids, combined with the *in vitro* findings of activity against multidrug-resistant pathogens causing STIs, suggest that lefamulin may be a potential treatment option for patients with STIs caused by these pathogens. As it is very difficult to measure the exposure of drugs at the sites of STIs and given the challenges in developing clinically relevant animal STI models (22), a translation of AUC/MIC ratios for dose selection from nonclinical animal model data to the clinical setting remains challenging. Tissue distribution data as collected in this study will support the selection of potential therapeutic doses for STI caused by different bacterial pathogens and involving a variety of tissues and glands of the male and female urogenital tract.

Safety and tolerability of lefamulin have been established in patients with CABP (12, 14). Clinical studies are therefore warranted to evaluate the efficacy of lefamulin in patients with STIs.

## MATERIALS AND METHODS

### Study design.

In this preclinical study, a single IV bolus dose (30 mg/kg) of [^14^C]-lefamulin was administered to male and female rats. All procedures were conducted in accordance with the standards set forth in the Code of Practice for the Housing and Care of Animals Bred, Supplied or Used for Scientific Purposes (23).

The study used 6 Sprague Dawley rats (3 males and 3 females; Charles River Ltd., Margate, Kent, UK). Animals were sexually mature at study initiation with males ranging between 7 to 10 weeks old and females 10 to 15 weeks old. All animals weighed 246 to 276 g at time of dosing, were housed in a climate-controlled environment (temperature 21 ± 2°C, 55% ± 10% humidity) that used a 12-h light/12-h dark cycle, and were given *ad libitum* access to water and a standard pelleted diet (RM1 [E] SQC; Special Diets Services, Witham, Essex, UK). Health status was monitored during a 4-day acclimatization period and throughout the study.

### Dose preparation and administration.

In an effort to evaluate a clinically relevant exposure, a lefamulin dose of 30 mg/kg was chosen for these analyses; based on previous work in Sprague Dawley rats, the 24-h area under the curve (AUC_0-24_) observed with a single 30 mg/kg dose was similar to a single 150 mg IV dose in humans (17). Therefore, [^14^C]-lefamulin (Pharmaron UK Ltd., Cardiff, UK) was prepared at a target dose level of 30 mg/kg (5 mL/kg) and 100 μCi/kg. An appropriate amount of radiolabeled lefamulin was added to nonradiolabeled lefamulin (DPx Fine Chemicals, Durham, NC, USA) in a prelabeled vial, and 20 mL of 0.9% saline (weight/volume; Sigma-Aldrich, Dorset, UK) was added in stages, dissolving the compound using sonication and magnetic stirring. Concentration, homogeneity, and radiochemical purity of the dose formulation were assessed before and after administering a single IV dose of 30 mg/kg. Concentration and homogeneity were assessed by removing replicate aliquots of dose formulation and using liquid scintillation counting to determine radioactive content. Radiochemical purity was assessed using high-performance liquid chromatography, using a Synergy 4μ Polar RP 80 Å 150 × 2 mm column (Phenomenex, Torrence, CA) with 0.1% formic acid (aq; Fisher Scientific, Loughborough, UK) or 0.1% formic acid in acetonitrile (Fisher Scientific) in the mobile phase, column temperature of 20°C, 210 nm UV wavelength, 1 mL/min flow rate, and running time of 30 min.

The lefamulin formulation was administered into a tail vein as a bolus injection, with the dose based on animal weight (obtained before dosing) and a dose volume of 5.0 mL/kg. Animals were not fasted at the time of dose administration.

### Specimen preparation and procedures.

At 0.5, 6, and 24 h post dose, 2 animals (1 male, 1 female) were euthanized using CO_2_ overdose and frozen. Frozen carcasses were set into blocks of aqueous carboxymethyl cellulose (1% wt/vol; Sigma-Aldrich, Gillingham, UK) and whole-body sagittal plane sections were obtained. Sections were collected at two levels of the rat carcass: the first (level 1) to investigate the distribution of radioactivity in the kidney (both male and female) and ovary/uterus (females only) and the second (level 2) to investigate the distribution of radioactivity in reproductive organs and tissues associated with renal elimination.

QWBA analyses were conducted based on procedures previously described (24); in brief, 30-μm sections were collected on autoradiography tape, freeze-dried, and exposed alongside radioactive [^14^C] microscale standards (American Radiolabeled Chemicals Inc., St. Louis, MO) against imaging plates at ambient temperature in the dark for 7 days. The distribution of radioactivity ([^14^C]-lefamulin) was determined using a Fuji FLA-5100 fluorescent image analyzing system (Fujifilm Life Sciences, Stamford, CT) with associated Tina version 2.09 (Elysia-Raytest, Straubenhardt, Germany) and SeeScan version 2.0 (LabLogic Systems Ltd., Sheffield, UK) software. For each exposure, a standard curve was produced in SeeScan using data from the autoradiographic microscale standards from which tissue concentrations of radioactivity (nCi/g) were determined.

MARG analyses were performed as previously described (25) and modified such that groups of tissues were evaluated *in situ* from sections of the frozen whole-body carcasses described above versus excision of individual organs/tissues for subsequent freezing and sectioning. Specifically, 10-μm tissue sections were collected onto tape, transferred to glass microscope slides using the CryoJane tape transfer system (Leica Biosystems Inc., Buffalo Grove, IL), air dried, and coated with nuclear emulsion. Slides were exposed in light-tight cassettes at −20°C for up to 6 weeks, then subjected to development and fixation procedures followed by hematoxylin and eosin (H&E) staining. Slides were inspected using an Axio Imager.A2 microscope (ZEISS Microscopy, Cambridge, UK) under both bright and dark field. Images were captured using the Retiga 2000R (Teledyne Photometrics, Tucson, AZ) digital camera. Interpretation of MARG results was purely qualitative and supported by images captured with the digital camera.

### Statistical analyses.

To evaluate the relative penetration of [^14^C]-lefamulin into relevant tissues, ratios of concentrations in the target tissue to concentrations in blood (tissue:blood) and lung (tissue:lung) were calculated at each individual time point for both male and female animals. The choice to include tissue:lung was made based the relative amount of clinical and pharmacokinetic data surrounding the lung compartment in patients and volunteers as a point of reference. In situations where concentrations in blood were below the limit of detection (i.e., <0.062 μg equivalents/g), the tissue:blood denominator was assumed to be a maximum of 0.062 μg equivalents/g.
